# Supplementary material for: An analysis of 45 large-scale wastewater sites in England to estimate SARS-CoV-2 community prevalence
Source: Nat Commun. 2022 Jul 25;13:4313. doi: 10.1038/s41467-022-31753-y (PMC9312315; doi:10.1038/s41467-022-31753-y)
Supplement: Supplementary file 1 — Supplementary Information [file 41467_2022_31753_MOESM1_ESM.pdf]

Supplementary Information for "An analysis of 45 large-scale wastewater sites in England to estimate SARS-CoV-2 community prevalence"

Supplementary Table S1: Wastewater data and meta-data. These data are used within the modelling to improve estimates of prevalence. Note that only time-varying variables are used in input of the random effect model as it already uses subregion-level coefficients and therefore does not benefit from any subregion-level metadata.

	**Raw Dataset**					**Aggregated dataset**		
**Variable**	Observations	% complete	Median value	25.00%	75.00%	Median value	25.00%	75.00%
**Sars-CoV-2**								
Sample concentration of SARS-CoV-2 in gene copies per litre	4863	100*	4371.3	235.2	18024	12114.1	3043.6	38913.4
Standard deviation of replicates	4863	100	5.6	0.2	18.6	14.1	4.8	43.3
Fraction of both samples below Limit of Quantification (LoQ)	4863	100	0.2	-	-	0.05	-	-
Fraction of both samples below Limit of Detection (LoD)	4863	100	0.3	-	-	0.08	-	-
Sample control volume	4863	100	2	2	2	2	2	2
**Biochemical covariates**								
Sample pH	471	9.7	7.6	7.4	7.8	7.6	7.5	7.7
Sample concentration of ammoniacal nitrogen	4308	88.6	30	21	40	27.9	20	36.3
Sample concentration of orthophosphates	4156	85.5	3.2	2.2	4.4	3.1	2.2	4.1
Sample concentration of suspended solids	4146	85.3	240	160	330	228.3	140	310
Method of sampling (composite samples or 'grab')	4863	100	0.54	-	-	0.6	-	-
Delay in hour between collection and lab reception	4852	99.8	23.5	22.2	25	23.8	22.5	25
**Demographic covariates**								
Population in subregion	-	-	-	-	-	493125	207747	1757742
Proportion coverage of subregion population	-	-	-	-	-	0.9	0.3	1.9
Catchment area covered, km2	-	-	-	-	-	318.6	149	555.3

* Note that this summary data is after removing the raw data observations where sars-cov-2 record was NA (n=1365)

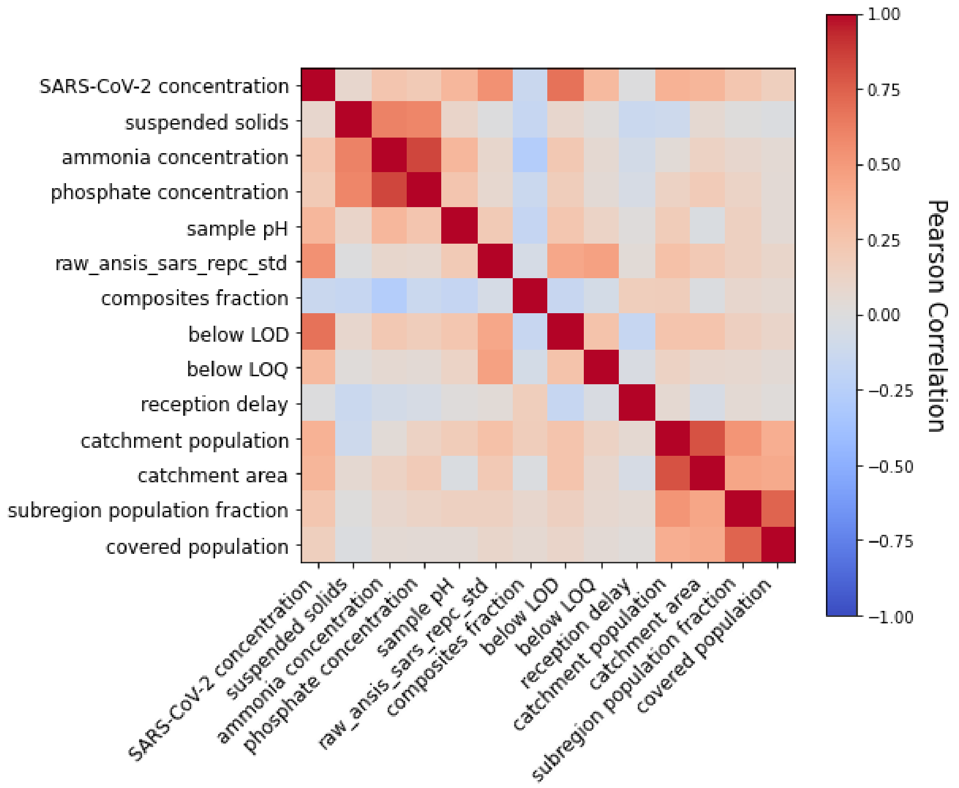

Fig. S1: Pearson correlation coefficients between variables considered in the model. Blue colours indicate negative correlations while red colours indicate positive linear correlation.

Supplementary Note 1. Full methods for Concentration and Quantification of SARS-CoV-2 RNA in Wastewater.

The protocols described within Farkas *et al.* and Walker are summarised below, following the MIQE guidelines and provided 'alongside' so the similarities and differences (underlined in the Walker column) are clearly indicated. All laboratory procedures are carried out in BLS2 Microbiological Safety Cabinets. In both methods the KingFisher Flex Purification System methods is used for RNA extraction, and the RT-qPCR step is the same.

Supplementary Table2. Side-by-side comparison of concentration and quantification of SARS-CoV-2 in Wastewater.

**Farkas**	**Walker**
Sampling from WW	
Sample consists of either a grab or composite sample, but there is no adjustment of methods after this point. Sample volume is 100-200ml of untreated WW. Sample is transported to the lab within 24 hrs, and chilled during transportation.<br>Each day include:<br>- Process negative control: 100-200 ml sterile water<br>- Process positive control: 90-150 ml sterile water spiked with 10^5 gc PRRSV<br>- Extraction negative control: 500 ml PBS<br>- Extraction positive control: 500 ml PBS spiked with 10^5 gc MNV	Sample volume is at least 150ml of untreated WW. Sample transportation is the sample as Farkas. Each day include:<br>- Process negative control: 100-200 ml sterile water<br>- Process positive control: 90-150 ml sterile water spiked with phi6, high enough in concentration that a 0.01% recovery can be quantified (> 10^3)<br>- Extraction negative control: 500 ml PBS<br>- Extraction positive control: 500 ml PBS spiked with 10^5 gc MNV
Concentration Step 1	
Centrifugation step 1 is to remove large particulate matter. Divide original sample into 50 ml centrifuge tubes. Centrifuge samples 3,000 x g at 4°C for 30 mins. Remove the supernatant and transfer to a new sterile 250 ml bottle. Adjust pH to 7-7.5 (with 1M NaOH) to enhance protein binding.	Include the entire 200 ml sample in a large centrifuge bottle. Centrifuge sample at 10,000 x g at 4oC for 30 mins. Decant 150 ∓ 1g of the clarified supernatant into clean bottles. To the sample and one of the negative controls, add the phi6 process control ($1 \mp 0.01\ ml$).
Concentration Step 2	
Add 1:3 ratio of 40% PEG 8000, 8% NaCl solution to reach a final concentration of 10% PEG 800, 2% NaCl. Incubate samples at 4°C for 14-18 hrs. Centrifuge at 10,000 x g for 30 min at 4°C. Discard supernatant, use the pellet in the further steps. Resuspend pellet in in 500 ml PBS. If RNA extraction is not to happen immediately prepare for medium or long term storage.	Pour the sample and the process control into centrifuge bottles containing 60 ∓ 1 g ammonium sulphate. Dissolve the ammonium sulphate by inverting several times. Incubate the samples at 3 ∓ 2 ^{o}C for 60-180 mins. Centrifuge the chilled samples at 10,000 x g for 30 mins at 4 ∓ 1 ^{o}C. Discard supernatant, use the pellet in further steps.
Nucleic acid extraction	
(Optional step to add 10^5 gc MNV as a positive control to each sample). Add 2 ml of NucliSens lysis buffer to 15ml centrifuge tube. Add 500 ul of sample and vortex. Incubate for 10 mins. Add 50 ul of well-mixed magnetic bead solution from the extraction kit and mix. Incubate (10 mins) and centrifuge for 2 min at 1500 x g. Wash with buffer and resuspend. Allow magnetic beads to settle, and repeat. Add 100 ul of elution buffer – the RNA material should transfer to this buffer and be used in qRT-PCR step.	Resuspend pellet in 2 ml of NucliSENS lysis buffer. Transfer the lysis buffer and pellet mixture to clean tubes, or 24 well deep-well plates. Incubate for 10 mins. Add 50 ul of well-mixed NucleiSENS magnetic bead solution from extraction kit and mix.
Reverse transcription	
N1 gene target, and PRRSV for positive control.	N1 gene target and phi6 for positive control.
SARS-CoV-2 RT- qPCR	
Prepare probes, noting that each sample should be run in duplicate. Prepare standard dilution series in	Prepare probes, noting that each sample should be run in duplicate. Prepare standard dilution series in

the range of $10^6 – 10^0$ gc/ul and a no template control. 5ul of the elution buffer is added to each qRT-PCR reaction mix. Carry out a one-step qRT-PCR reaction, cycling through 95ºC and 60/65ºC cycles 45 times. The qRT-PCR returns the number of cycles where the N1 gene has been amplified. The (geometric) mean are taken from duplicate samples. Ct values >40 should be considered negative.	the range of $10^6 – 10^1$ gc/ul and a no template control. 5ul of the elution buffer is added to each qRT-PCR reaction mix. Carry out a one-step qRT-PCR reaction, cycling through 95ºC and 60/65ºC cycles 45 times.
Conversion of Ct values to viral genome copies per litre	
The standard dilution series provide the "intercept" and "slope" for the assumed $\log_{10}$ linear relationship between Ct and gc per sample. The exact values may vary from sample to sample but should have limited variability across the time series.<br><br>The estimate of N1 gc per ml WW is calculated as follows:<br><br>= gc per qRT-PCR reaction x 20 / sample supernatant volume (ml)	The standard dilution series provide the "intercept" and "slope" for the assumed $\log_{10}$ linear relationship between Ct and gc per sample. Report the coefficient of determination (R^2).<br><br>Standard curves with $R^2<0.98$ and the slope not between -3.6 and -3.1 (equivalent of 90% - 110% amplification efficiency) should not be used to calculate results. Recalculate the standard curves, and potentially the plates.<br><br>Phi6 recovery:<br>ΔC_q= Cq (sample RNA) – Cq (phi6 positive control)<br>m = slope of the phi6 standard curve<br>Phi6 process control recovery, $P = 10^{\Delta C_q/m}\ x\ 100\%$<br><br>SARS-CoV-2 estimation:<br>ΔC_q= Cq (sample RNA) – standard curve intercept<br>m = slope of the ssRNA standard curve<br>RNA in sample, G gc/ul:<br>$G = 10^{\Delta C_q}$<br><br>The sample WW is typically carried out in replicate, so the geometric mean on the 2 samples are used in calculations. If the individual values differ by <50% of the mean this indicates variability beyond what is expected and should be investigated.

Supplementary Table S3: A list of primers and probes used for RT-qPCR (written 5' to 3')

Target	Name	Type	Sequence	Reference
SARS-CoV-2 N gene	2019-nCoV_N1-F	Forward primer	GAC CCC AAA ATC AGC GAA AT	(CDC, 2020) nCOV_N1 Forward Primer Aliquot, Cat no 10006821
SARS-CoV-2 N gene	2019-nCoV_N1-R	Reverse primer	TCT GGT TAC TGC CAG TTG AAT CTG	(CDC, 2020) nCOV_N1 Reverse Primer Aliquot, Cat no 10006822
SARS-CoV-2 N gene	2019-nCoV_N1-P	Probe	ACC CCG CAT TAC GTT TGG TGG ACC	(CDC, 2020)
Phi6 *6S_1* gene	phi6_forward	Forward primer	TGG CGG CGG TCA AGA GC	(Gendron *et al.*, 2010)

Phi6 *6S_1* gene	phi6_reverse	Reverse primer	GGA TGA TTC TCC AGA AGC TGC TG	(Gendron *et al.*, 2010)
Phi6 *6S_1* gene	phi6_probe	Probe	CGG TCG TCG CAG GTC TGA CAC TCG C	(Gendron *et al.*, 2010)

For the period Feb 2021 to Mar 2022, below we provide the summary of standard dilution series metrics for approximately 1,821 plates used to generate the data (for the N1 gene only).

Supplementary Table S3. Standard dilution metrics for plates used to quantify SARS-CoV-2 in wastewater

	Slope	Efficiency	R^2	Y-intercept
**Mean**	-3.36	98.70	0.99	41.91
**Median**	-3.35	98.78	0.99	41.78
**%CV**	3.43	4.73	0.45	2.40
**Max**	-3.10	109.99	1.00	46.51
**Min**	-3.59	90.00	0.98	38.51

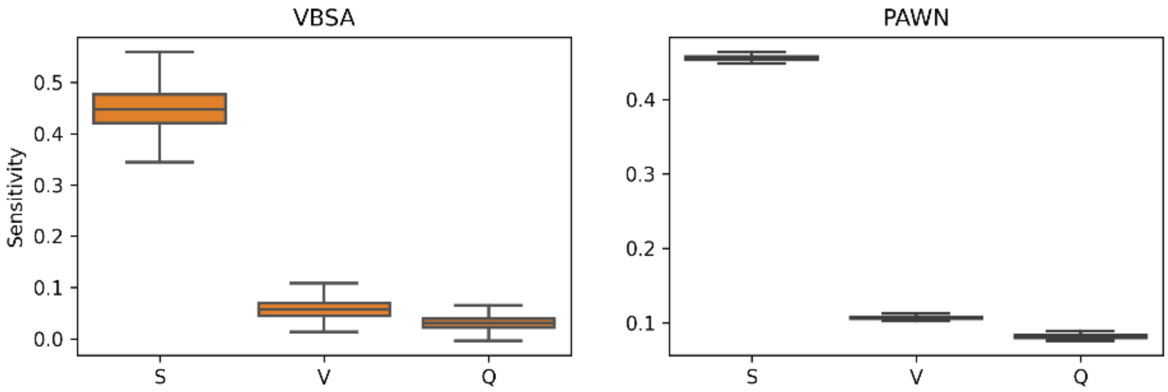

Fig. S2: Sensitivity analysis showing that better knowledge of mean viral concentration in stool (*S*) is more important than other parameters (volume (*V)* and flow rate (*Q*)) for constraint of prevalence estimates in back-calculation (using VBSA (left) and PAWN (right) methods). Using VBSA and PAWN, with 10,000 samples and 1,000 bootstraps, at concentration *C* = 10,000 gc/L. Parameter values are sampled from the uncertainty distributions.

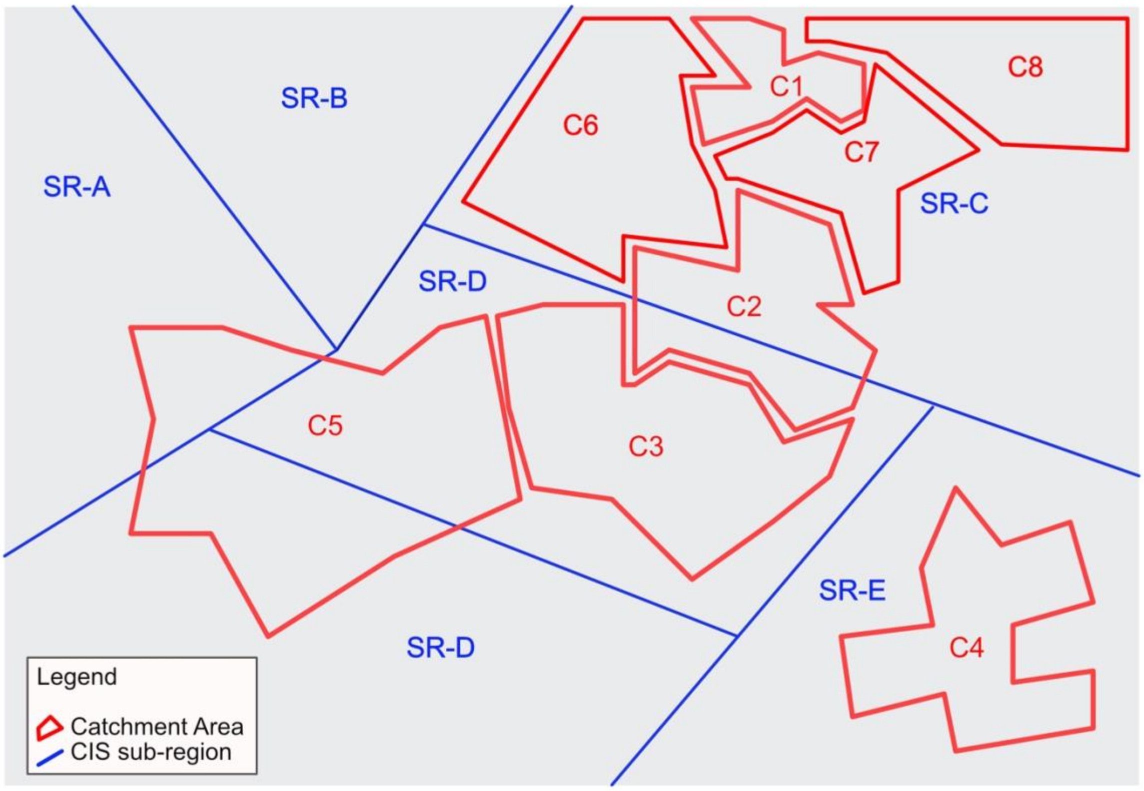

Fig.S3: Schematic of the mapping of wastewater catchment areas (red polygons) to Covid Infection Survey (CIS) sub-regions. Within sub-region E (SR-E) only one catchment (C4) is sampled; in this simple example the catchment samples only wastewater from that sub-region. Within sub-region D (SR-D), catchment C3 is a full sample of the sub-region while C5 and C2 have a portion of the catchment within sub-region D. For estimation of the SARS-CoV-2 prevalence for catchment SR-D, the model estimates from catchments C2, C3, and C5 are combined using a weighting according to the proportion of the catchment within the sub-region.

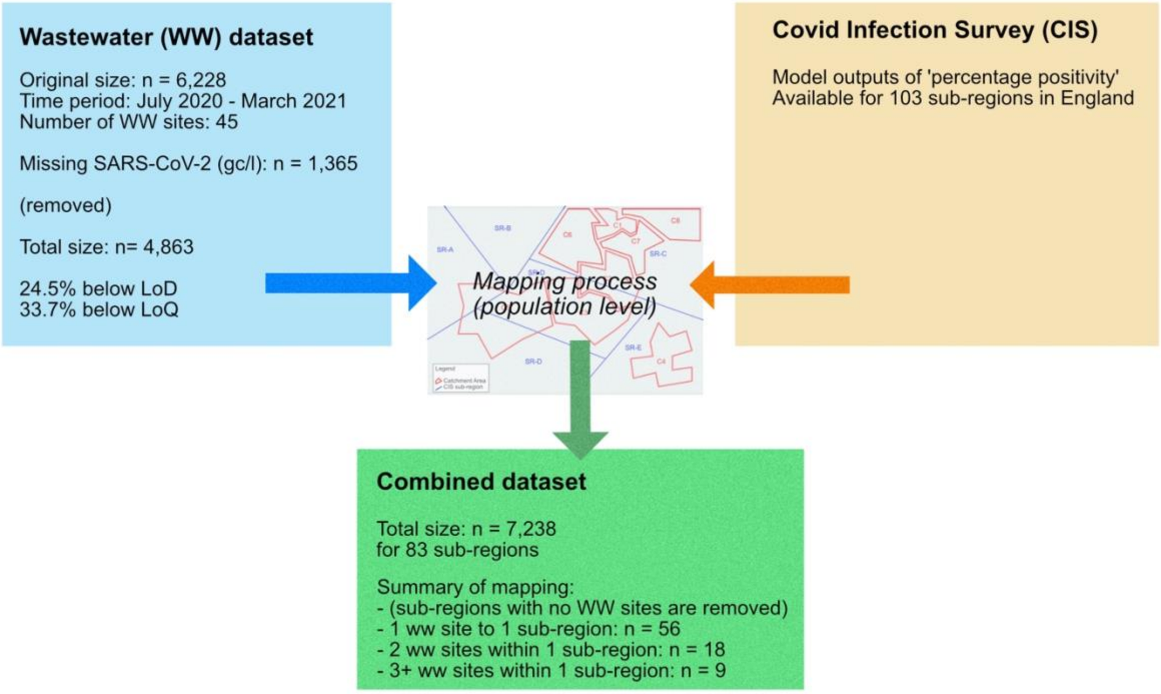

Fig. S4: Schematic of the process used to compare the wastewater dataset to the positivity data from the Covid infection survey.

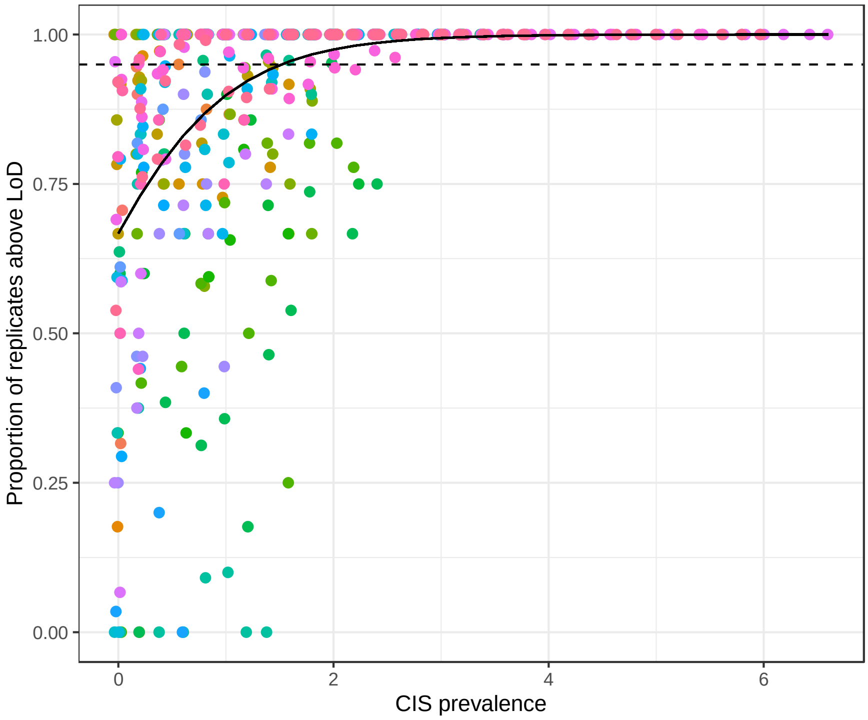

-

Fig. S5. Comparison of the site-level proportion of WW samples that are above LoD for grouped values of mapped CIS positivity. Coloured circles indicate the proportion of samples above LoD for each site at the corresponding prevalence. The black line indicates the (logistic) model fit of all the observations, and the dashed line indicate 0.95; where the black line intersects this may be regarded as a field LoD.

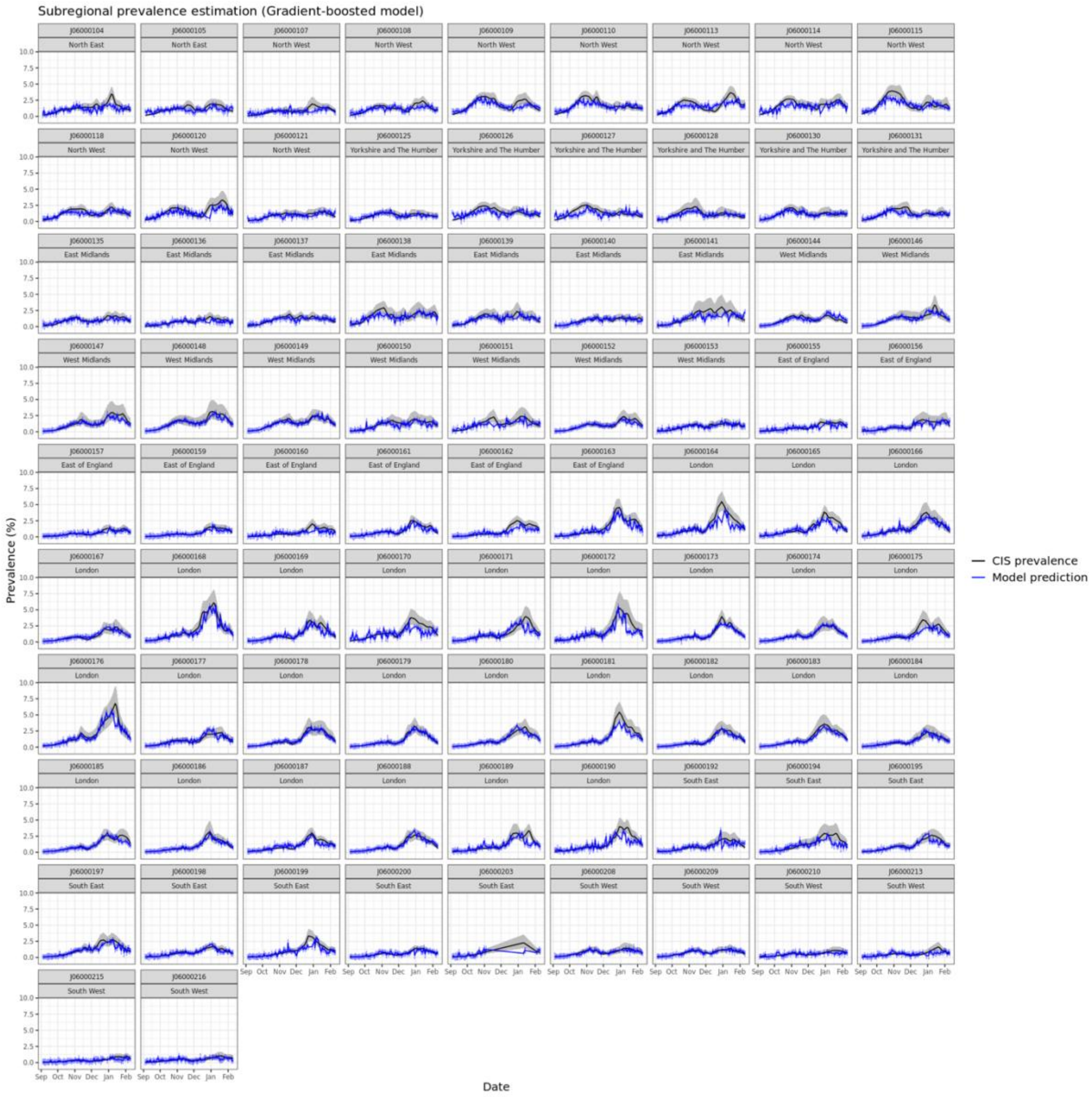

Fig. S6 Model prediction from the Extreme Gradient Boosted (XGB) model and CIS prevalence for the 83 site/CIS pairs at a sub-regional level.

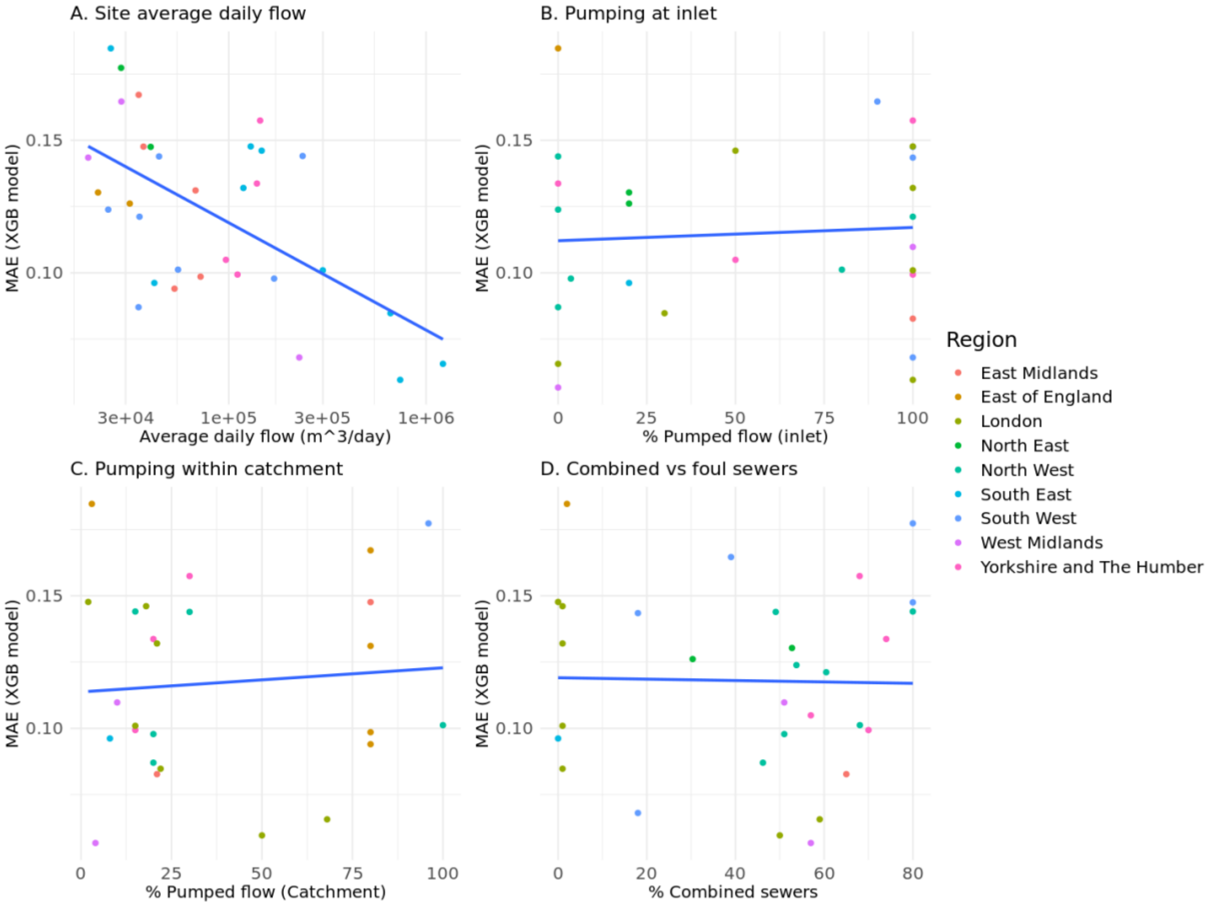

Fig. S7 Model performance at each WTP site depending on network characteristics provided by water companies. A site-level MAE was calculated by averaging, weighting by population covered, the MAE of the CIS subregions included in the catchment, and plotted across sites characteristics. The region of each site is indicated by the color of the dots, with the blue line representing the linear regression between score and site characteristics. None of these regressions yielded a significant result (p>0.05) when included in a linear model (site score ~ site characteristic).

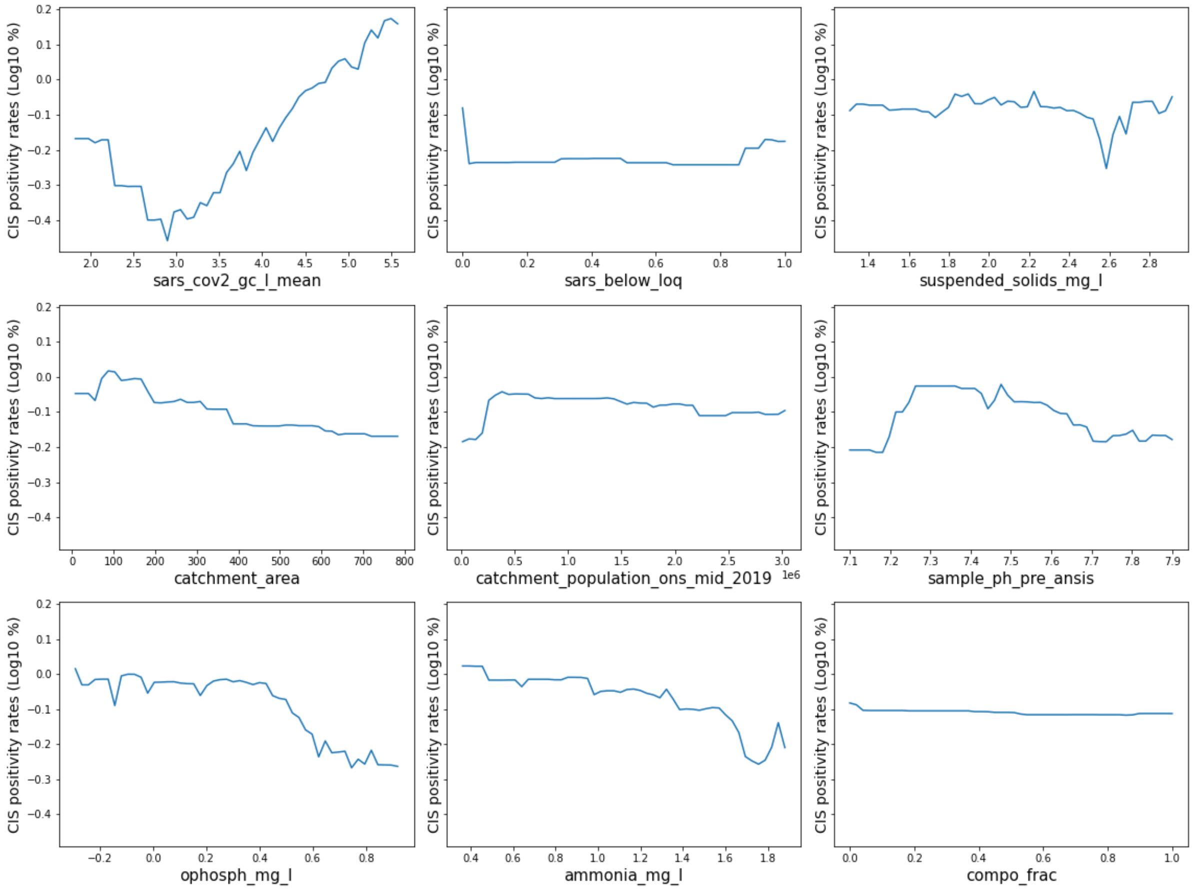

Fig. S8 Partial dependency plots extracted from the XGB model trained on the whole dataset.

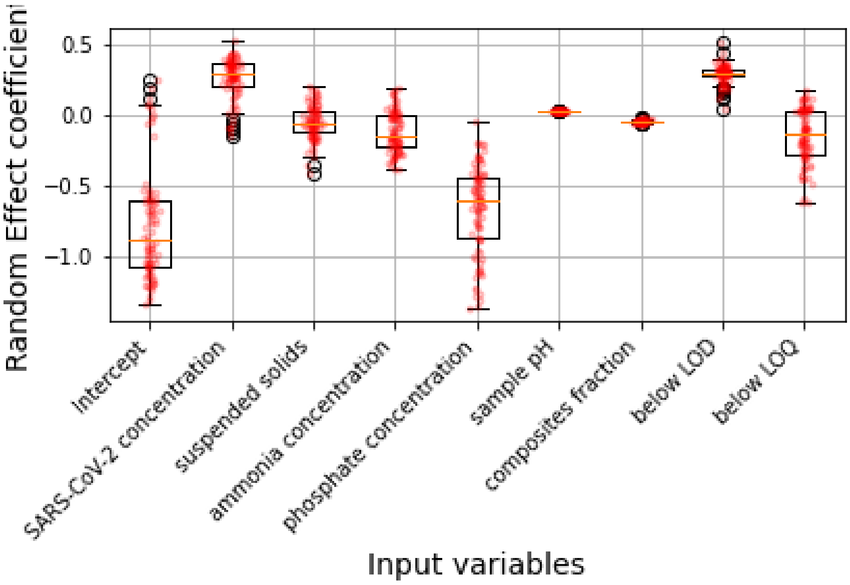

Fig. S9 Random effect intercepts (first column) for the random effects model and coefficients after fitting the whole WW/CIS for each of the 83 CIS subregions. Lower and upper hinges of the box plots correspond to first and third quartile with middle line corresponding to the median.

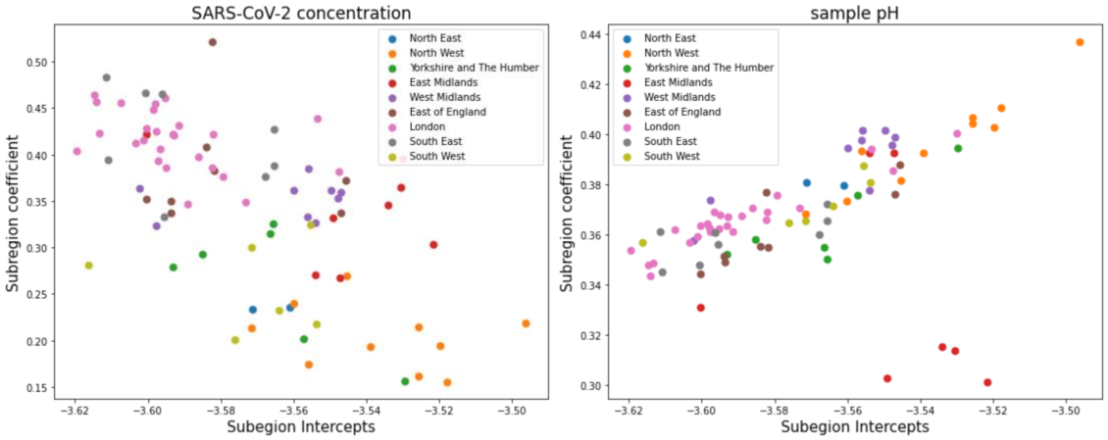

Fig. S10 Scatter plots of random effects for the two variables most correlated with the subregions' intercepts: SARS-CoV-2 concentration (left) and sample pH (right). In addition, subregional points have been coloured by region to shed light on the geographic clustering which naturally emerges from these representations.

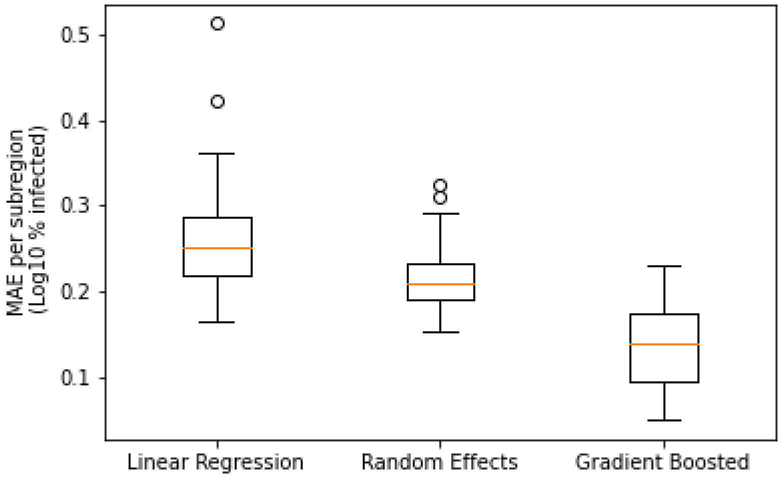

Fig. S11 Models performance measured by their residual error per subregion. Lower and upper hinges of the box plot corresponds to first and third quartile with middle line corresponding to the median.

References:

CDC (2020) 'Research Use Only 2019-Novel Coronavirus (2019-nCoV) Real-time RT-PCR Primers and Probes'. Available at: https://www.cdc.gov/coronavirus/2019-ncov/lab/rt-pcr-panel-primer-probes.html (Accessed: 21 June 2021).

Gendron, L. *et al.* (2010) 'Evaluation of Filters for the Sampling and Quantification of RNA Phage Aerosols', *Aerosol Science and Technology*, 44(10), pp. 893–901. doi: 10.1080/02786826.2010.501351.
